# Exploring the structural and construct validity of the Brazilian version of the Eating Pathology Symptoms Inventory

**DOI:** 10.1590/1806-9282.20241894

**Published:** 2025-06-02

**Authors:** João Paulo Lessa, Maíra Stivaleti Colombarolli, Jônatas de Oliveira

**Affiliations:** 1 Universidade Tuiuti do Paraná, Graduate Program in Forensic Psychology – Curitiba (PR), Brazil.; 2 Institute for Research on Behavior and Food – Curitiba (PR), Brazil.; 3 Universidade de São Paulo, Faculty of Philosophy, Sciences and Letters of Ribeirão Preto – Ribeirão Preto (SP), Brazil.; 4 Universidade de São Paulo, School of Medicine, Faculty of Medicine – Sao Paulo (SP), Brazil.

**Keywords:** Binge-eating disorder, Feeding and eating disorders, Feeding behavior, Craving, Psychometrics

## Abstract

**OBJECTIVE:**

The aim of this study was to evaluate the psychometric properties of the Eating Pathology Symptoms Inventory within the Brazilian context. Specifically, it assessed the scale's efficacy in measuring disordered eating behaviors, with an emphasis on binge eating, and investigated its relationship with food cravings.

**METHODS:**

This cross-sectional study utilized data from a non-probabilistic convenience sample of 1,374 Brazilians. Confirmatory factor analysis and multiple-group confirmatory factor analysis were employed to examine the factor structure and assess invariance across groups with and without binge-eating symptoms. Reliability was evaluated using Cronbach's alpha and McDonald's omega, while external validity was assessed through Pearson's correlations between Eating Pathology Symptoms Inventory and Food Cravings Questionnaire-Trait-reduced scores. Discriminant validity was analyzed using Welch's t-test.

**RESULTS:**

The confirmatory factor analysis supported the eight-factor model, demonstrating a good fit across the overall sample (Comparative Fit Index=0.936, root mean square error of approximation=0.057). Reliability analysis indicated high internal consistency, with Cronbach's alpha and McDonald's omega values ranging from 0.745 to 0.917. Multiple-group confirmatory factor analysis confirmed measurement invariance across groups with and without binge-eating symptoms. The Welch two-sample t-test showed significantly higher Eating Pathology Symptoms Inventory scores in the binge-eating group. Furthermore, Eating Pathology Symptoms Inventory subscales related to binge-eating behaviors exhibited strong correlations with food craving scores, supporting the scale's external validity.

**CONCLUSION:**

The Eating Pathology Symptoms Inventory demonstrates strong reliability and validity as a tool for assessing eating disorder symptoms in Brazil, with effectiveness in distinguishing binge-eating behaviors.

## INTRODUCTION

The presentation of eating disorders (EDs) in the contemporary era warrants consideration of atypical anorexia nervosa, the potential occurrence of binge-eating behavior in individuals with orthorexia nervosa, and the assessment of muscle building in EDs. Given the gap and the need for innovative methods to comprehend and assess ED symptoms, Forbush et al. generated substantial evidence validating the Eating Pathology Symptoms Inventory (EPSI)^1,2^. It is a multidimensional self-report measure developed to assess ED pathology among a wide variety of populations^2^. This scale innovatively differentiates between cognitive restraint and food restriction, domains that are related to eating behaviors such as binge eating and are influenced by food cravings (FCs). Currently, these domains are measured by the Food Cravings Questionnaire-Trait (FCQ-T), which can differentiate between various ED categories^3^.

FCs are not merely physiological or behavioral responses but a multifaceted phenomenon. They involve specific motivations manifested through persistent thoughts, vivid sensations, and mental imagery that drive the pursuit and consumption of food for pleasure or relief^4^. These cravings are often identified as precursors to binge eating, characterized by consuming large quantities of food in a short period accompanied by a perceived loss of control. Research has shown that both trait and state FCs can predict the overall intensity of cravings experienced during virtual reality exposure in individuals with EDs^5^.

Studies on the EPSI have yielded corroborating evidence of its construct validity and internal structure, as demonstrated by invariance analysis across gender, age, and minority groups^6-8^. It is thus important to examine the factor structure within the Brazilian population and to ascertain evidence that the scale can reliably assess different groups. Furthermore, it is essential to gain insights into the relationship between FCs and eating pathology to determine how a multidimensional instrument can be used to distinguish binge-eating disorders across various groups. The present study evaluates the psychometric properties of the EPSI in relation to binge-eating symptoms to ensure its reliability and validity for assessing ED symptoms in this population. These analyses aim to deepen the understanding of how different groups manifest behaviors related to ED, thereby enhancing the tool's applicability and effectiveness within the Brazilian context.

## METHODS

### Participants

A non-probabilistic convenience sample comprising 1,374 Brazilian participants aged 18–67 years was recruited from the general population through online advertisements disseminated via social media platforms (July–September 2023). Eligibility criteria included being aged 18 years or older, identifying as Brazilian (residing either within the country or abroad), and having the capacity to provide informed consent. Individuals were excluded if they were unable to complete the survey due to language barriers or cognitive impairments. Throughout the questionnaire, attention-check items, such as "what is 5+7?", were included to ensure that participants were actively engaged while completing the survey. Participants who failed these attention-check questions were excluded from the initial dataset. To enhance participation, a snowball sampling method was employed, whereby the participants were encouraged to invite peers to take part in the study (excluded cases=15). The sample size was determined based on methodological guidelines for confirmatory factor analysis (CFA), ensuring sufficient participants per parameter. As part of a larger study, this sample also allows for the exploration of the scales’ psychometric properties, meeting the objectives of both the broader project and this research. The participants had a mean age of 32.7 years (standard deviation [SD]=10.35); 40% reported being full-time students, and 76.5% identified as Caucasian. Ethical approval for the study was granted (CAAE: 9232123.6.0000.5146), and informed consent was obtained from all participants prior to their inclusion in the study.

### Measures

#### Screening questionnaire for disordered eating

This instrument evaluates the occurrence of binge-eating episodes and compensatory behaviors over the past 3 months^9^, with responses categorized as "not at all," "less often than once a week," "once a week," or "two or more times a week." In the Brazilian context, this questionnaire has shown good internal consistency and has been effectively integrated into an etiological model of disordered eating behaviors among Brazilian women^10,11^. We employed a dichotomous classification of symptoms (control group vs. binge-eating group), categorizing individuals as symptomatic if they exhibited a higher frequency of ED behaviors, in contrast to those who displayed no such symptoms.

#### Food Cravings Questionnaire-Trait-reduced

The Brazilian version of the Food Cravings Questionnaire-Trait-reduced (FCQ-T-r) was used to assess the frequency and intensity of FCs^12^. The FCQ-T-r is a 15-item instrument designed as an abbreviated version of the original Food Cravings Questionnaire-Trait (FCQ-T). This shortened version preserves the comprehensive scope of the original scale while offering a more concise format, facilitating its application and interpretation in both research and clinical contexts. In the Brazilian study, the FCQ-T-r demonstrated excellent internal consistency, with a Cronbach's alpha of 0.95.

#### The Eating Pathology Symptoms Inventory

The EPSI is an assessment tool developed to meet the need for a contemporary measure of ED symptoms^1^. Originally proposed by Forbush et al., the instrument comprises 45 items and evaluates 8 key factors essential for understanding ED symptomatology: body dissatisfaction, binge eating, cognitive restraint, overexercising, restricting, purging, muscularity-oriented behaviors, and negative attitudes toward obesity^1^. The Brazilian version of the questionnaire is now fully prepared and readily available for use, offering a valuable tool for future research and clinical applications^2^.

### Statistical analysis

All analyses were conducted in the R environment using the lavaan package. The chi-square test was employed to assess whether there were significant differences in demographic and behavioral variables between the group with ED symptoms and the control group. CFA and multiple-group CFA were employed to examine the psychometric properties of the measurement model and investigate group differences in EPSI, following the fit indices^13,14^. Reliability indices, such as Cronbach's alpha and McDonald's omega, were calculated to assess the internal consistency of EPSI. External validity was evaluated using Pearson's correlation with variables, such as EPSI (total scores and subscale scores) and FCQ-T-r. Discriminant validity was assessed using Welch's t-test between groups classified from Screening questionnaire for Disordered eating due to unequal variances.

## RESULTS

A total of 1,374 individuals participated in the study, comprising 1,192 women (86.7%), 173 men (12.5%), and 9 non-binary individuals (1%). The average age of the participants was 32 years (SD=10.35, range: 18–67), with a mean body mass index (BMI) of 26.40 kg/m² (SD=6.29, range: 14.2–55.33). The sample included some cases of underweight, with 77 individuals (5.6%) classified as such according to the BMI criteria. Nearly half of the participants had a normal weight (n=592, 43%). Cases of overweight were reported in 345 participants (25.1%), and obesity was observed in the same number (n=345, 25.1%). Additionally, 15 participants (1.0%) were unable to specify their weight. There were no statistically significant differences observed across the various variables analyzed. Regarding BMI (χ²=764/809, p=0.124), a higher proportion of individuals in the symptom group were classified as obese (44%), whereas the control group predominantly exhibited normal weight (48%). Concerning student status (χ²=1/0.78, p=0.375), the majority of participants in both groups were not students (symptom group and control group=61 and 59%, respectively). Ethnic distributions also did not differ statistically significantly (χ²=4/2.56, p=0.633), with light-skinned individuals comprising the majority in both groups (73% in the symptom group and 77% in the control group). Regarding cigarette use (χ²=3/5.71, p=0.127), most participants reported no smoking (91 and 95% in the symptom group and control group, respectively). Patterns of alcohol consumption were similarly distributed (χ²=4/3.95, p=0.41). Finally, income analysis (χ²=5/6.37, p=0.27) revealed that the most frequent income range was 1–3 minimum wages (33% in the symptom group and 30% in the control group). These findings indicate that no significant differences were observed between the groups in the analyzed variables.

### Eating Pathology Symptoms Inventory confirmatory factor analysis

To evaluate the fit of the eight-factor model previously validated in the literature, CFA was performed^4-6^. The model demonstrated a good fit for the overall sample, including both binge-eating and control participants (χ²=5,049.437[917], p<0.001; Comparative Fit Index [CFI]=0.936; Tucker-Lewis Index [TLI]=0.931; root mean square error of approximation [RMSEA]=0.057 [p<0.001]; Standardized Root Mean Square Residual [SMRM]=0.066). Factor loadings ranged from 0.513 to 0.963, with Cronbach's alpha from 0.745 to 0.914 and McDonald's omega from 0.762 to 0.917. A multiple-group CFA was conducted to evaluate model consistency between groups with and without a binge-eating diagnosis. This approach tested nested models for configural, metric, and scalar invariance. As shown in Table 1, the results indicated strong factor invariance for the EPSI across these groups, evidenced by minimal changes in the CFI and TLI indices (<0.01) and RMSEA values (<0.005)^11,12^.

**Table 1:** Multiple-group analysis results.

	χ^2^	df	CFI	TLI	RMSEA
Configural	4,802.323	1,834	0.941	0.936	0.049
Metric	4,608.714	1,871	0.945	0.942	0.046
Scalar	4,961.282	1,998	0.941	0.941	0.046

Note: RMSEA: root mean square error of approximation; CFI: Comparative Fit Index; TLI: Tucker-Lewis Index; df: degrees of freedom; χ^2^: chi-square. All models were employed using WLSMV estimator in lavaan.

The Welch two-sample t-test revealed a statistically significant difference in EPSI total scores between the control and binge-eating groups (t=-17.092, degrees of freedom [df]=536.98, p<0.001). Individuals with binge-eating behaviors had higher EPSI total scores (mean [M]=82.17) compared to the controls (M=56.83), with a 95% confidence interval of −28.26 to −22.43. These results underscore the ability of EPSI scores to distinguish between eating behaviors, highlighting the significance of group differences in this study.

The external validity of the EPSI was evaluated using Pearson's correlation between correlation between EPSI total scores and its subscale scores and the FCQ-T-r total score and its subscale scores. The analysis revealed no significant correlations between EPSI subscales scores 5 and 8 with the FCQ-T-r total scores and its subscales scores. EPSI subscale score 2 exhibited the strongest correlation with the FCQ-T-r total scores and its subscales scores (r=0.593–0.808, p<0.001), indicating a robust relationship. In contrast, EPSI subscale score 6 showed the lowest correlation with the FCQ-T-r total scores and its subscales scores (r=0.079–0.231, p<0.001). Additionally, the EPSI total score and subscales scores 1, 3, 4, and 7 demonstrated mild correlation values with the FCQ-T-r total score and its subscales scores (r=0.235–0.646, p<0.001), as demonstrated in Table 2.

**Table 2:** Correlations of eating pathology symptoms and food cravings.

	1	2	3	4	5	6	7	8	9	10	11	12	13	14	15
1. EPSI total	–														
2. EPSI BD	0.739***	–													
3. EPSI BE	0.733**	0.590***	–												
4. EPSI CR	0.665***	0.410***	0.340***	–											
5. EPSI P	0.715***	0.506***	0.510***	0.449***	–										
6. EPSI R	0.398***	0.193***	0.038	0.210***	0.293***	–									
7. EPSI EE	0.612***	0.215***	0.246***	0.536***	0.289***	0.170***	–								
8. EPSI NO	0.571***	0.340***	0.393***	0.335***	0.337***	0.078**	0.217***	–							
9. EPSI MB	0.366***	0.021	0.046	0.250***	0.050	0.077**	0.521***	0.107***	–						
10. FC total	0.642***	0.587***	0.808***	0.337***	0.468***	0.014	0.188***	0.393***	-0.004	–					
11. FC LC	0.608***	0.542***	0.794***	0.300***	0.438***	-0.001	0.179***	0.388***	-0.012	0.933***	–				
12. FC PLN	0.480***	0.442***	0.593***	0.242***	0.349***	0.064**	0.137***	0.257***	0.004	0.805***	0.671***	–			
13. FC TT	0.646***	0.566***	0.753***	0.385***	0.478***	0.038	0.231***	0.409***	0.014	0.938***	0.845***	0.703***	–		
14. FC EM	0.473***	0.488***	0.642***	0.235***	0.344***	-0.031	0.079**	0.272***	-0.023	0.795***	0.683***	0.574***	0.656***	–	
15. FC C	0.457***	0.457***	0.652***	0.196***	0.320***	-0.107***	0.108***	0.295***	-0.010	0.769***	0.711***	0.575***	0.640***	0.652***	–

Note:

** p<0.05;

*** p<0.001. EPSI: Eating Pathology Symptoms Inventory; FCQ-T-r: Food Cravings Questionnaire-Trait-reduced; EPSI total: total score for EPSI; EPSI BD: EPSI subscale body dissatisfaction; EPSI BE: EPSI subscale binge eating; EPSI CR: EPSI subscale cognitive restraint; EPSI P: EPSI subscale purging; EPSI R: EPSI subscale restricting; EPSI EE: EPSI subscale excessive exercising; EPSI NO: EPSI subscale negative attitudes toward obesity; EPSI MB: EPSI subscale muscle building; FC total: food craving total score; FC LC: FCQ-T-r subscale lack of control; FC PLN: FCQ-T-r subscale intention and planning to eat; FC TT: FCQ-T-r subscale thoughts about food; FC EM: FCQ-T-r subscale emotions; FC C: FCQ-T-r subscale cues.

## DISCUSSION

This study investigates the psychometric properties of the EPSI within the Brazilian context. The results support the validity of the eight-factor model, demonstrating a strong fit across the overall sample, which includes individuals exhibiting binge-eating behaviors as well as those from the general population. Reliability coefficients confirm that the EPSI is a consistent and dependable tool for assessing ED symptoms across diverse populations, aligning with findings from previous research^6-8^.

Racine^15^ conducted the first study to investigate the association between domains of the EPSI and FC. A sample of 203 female participants were invited to rate high- and low-calorie food images on valence, arousal, and craving. The findings revealed that cognitive restraint was associated with increased pleasure and craving for low-calorie foods, while dietary restriction was linked to reduced pleasure and craving for both food types. These results highlight distinct patterns between cognitive restraint and dietary restriction and demonstrate the EPSI's effectiveness in capturing these differences. Our study represents the second investigation in the literature to explore these associations, further supporting the relevance of the EPSI in understanding FC dynamics.

The demographic and clinical characteristics of the sample enhance the study's validity by demonstrating minimal differences in non-clinical variables, such as BMI, student status, ethnicity, smoking habits, alcohol consumption, and income, between groups. This homogeneity reduces the influence of confounding factors. The predominance of women in the sample reflects trends in eating behavior research but limits generalizability to male and non-binary populations. Additionally, the multiple-group CFA confirmed the model's invariance across groups with and without a diagnosis of binge eating, suggesting that the EPSI reliably evaluates ED behaviors across diverse populations. Given the established invariance and the observed differences between these groups, the findings suggest that the EPSI is a promising tool for assessing disorders in the Brazilian context.

Moreover, the restriction and muscle-building subscales of the EPSI showed no significant association with FCs, indicating a lack of plausible connection between these factors. These results align with previous literature, as these subscales have shown minimal correlation with other ED measures^7,8^. The binge-eating subscale demonstrated the strongest correlations with FC scales and the total score, highlighting its critical role in assessing compulsive eating tendencies. FCs are frequently recognized as precursors to binge eating, and FC measures like FCQ-T have been shown to predict the intensity of cravings experienced during virtual reality exposure^5^. However, further validation in samples with a clinical diagnosis of ED is necessary to confirm its utility across diverse settings.

This study confirms the initial exploratory findings that the EPSI is a reliable and valid instrument for assessing disordered eating behaviors, particularly in relation to compulsive eating, within the Brazilian context. Its psychometric properties demonstrated the ability to differentiate individuals with and without patterns of disordered eating, reinforcing its broad applicability.

However, a notable limitation is the exclusion of other subtypes, such as bulimia nervosa and anorexia nervosa, from the invariance analysis due to the low number of participants identified by the screening questionnaire. This limitation restricts the generalizability of the findings to these specific populations. Additionally, reliance on self-reported data may have introduced recall bias or social desirability bias, potentially affecting the accuracy of responses. Future research should address these limitations by recruiting larger and more diverse samples, including sufficient representation of different subtypes of EDs. Longitudinal studies would also contribute to an in-depth understanding of the stability and predictive validity of the EPSI over time. Furthermore, exploring cultural and socioeconomic factors that may influence eating behaviors in the Brazilian context could provide additional insights. Despite these limitations, the current study offers valuable evidence supporting the psychometric robustness of the EPSI, contributing to the advancement of tools available for identifying and understanding disordered eating behaviors in diverse populations.

## ETHICAL STATEMENT

The appropriate informed consent was obtained, and the study was approved by the São Paulo University's Ethics Board under the approval number: CAAE: 69232123.6.0000.5146.
